# Supplementary material for: Variation in the Early Life and Adult Intestinal Microbiome of Intra-Uterine Growth Restricted Rat Offspring Exposed to a High Fat and Fructose Diet
Source: Nutrients. 2023 Jan 1;15(1):217. doi: 10.3390/nu15010217 (PMC9824396; doi:10.3390/nu15010217)
Supplement: Supplementary file 1 [file nutrients-15-00217-s001.zip › nutrients-2116628-supplementary.pdf]

Supplementary figures and tables

Variation of the Intestinal Microbiome during the life course of the Intra-Uterine Growth Restricted Rat Offspring Exposed to a High Fat and Fructose Diet

Liesbeth V. Maggiotto[1⊥], Shubhamoy Ghosh[1⊥], Bo-Chul Shin[1], Amit Ganguly[1], Venu Lagishetty [2,3], Jonathan P. Jacobs [2,3] and Sherin U. Devaskar[1]*

[1]Department of Pediatrics, Division of Neonatology & Developmental Biology, and UCLA Children's Discovery & Innovation Institute, [2]The Vatche and Tamar Manoukian Division of Digestive Diseases, [1,2]David Geffen School of Medicine at UCLA , Los Angeles, CA 90095-1752

[3]Division of Gastroenterology, Hepatology and Parenteral Nutrition, Veterans Affairs Greater Los Angeles Healthcare System, Los Angeles, CA.

Supplementary Figure 1

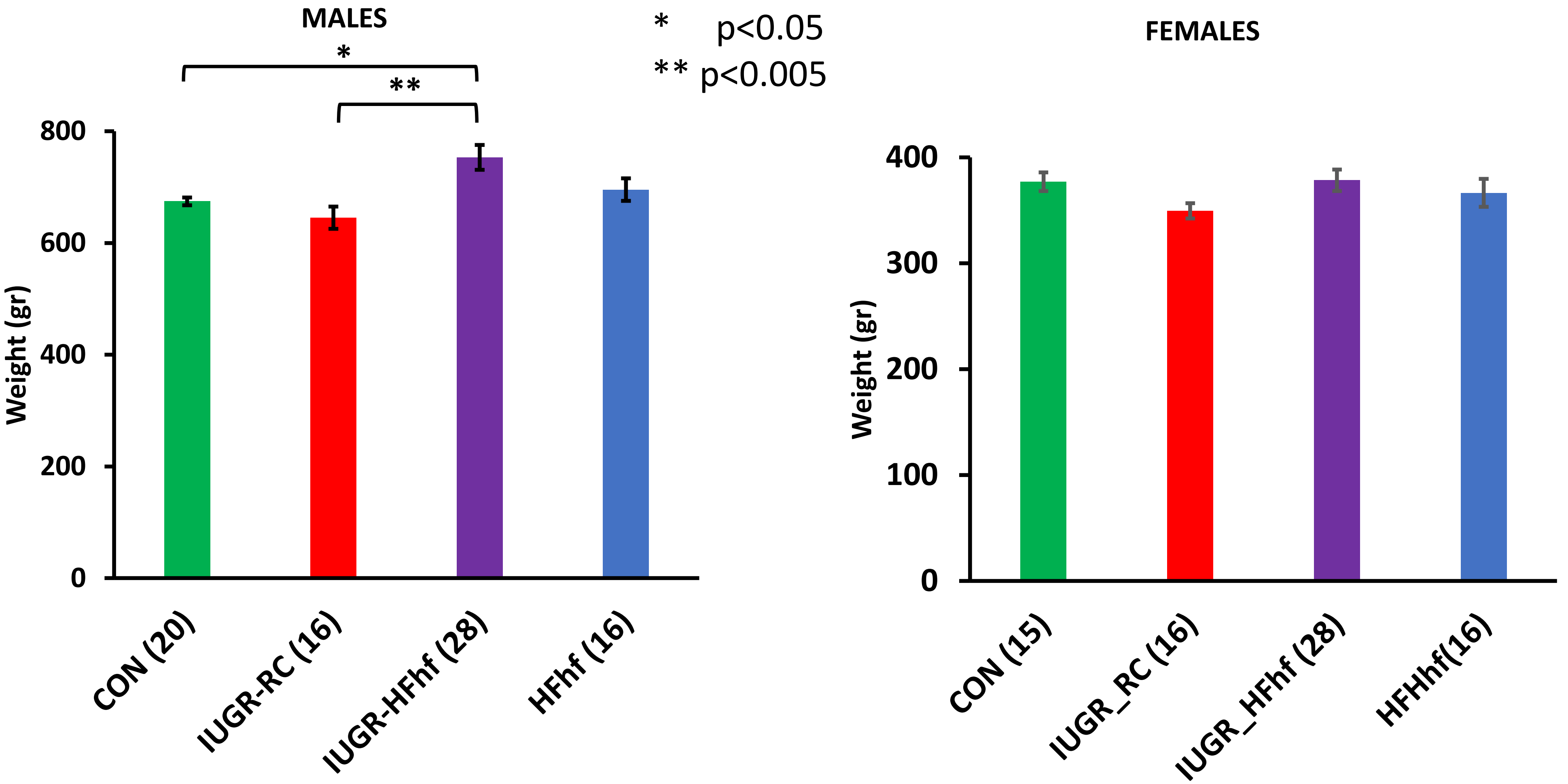

Bar graph showing bodyweights of 6 month old animals (n) from the four experimental groups. While males demonstrate significant differences (ANOVA and Tukey's test), females show a similar trend.

Supplementary Figure 2

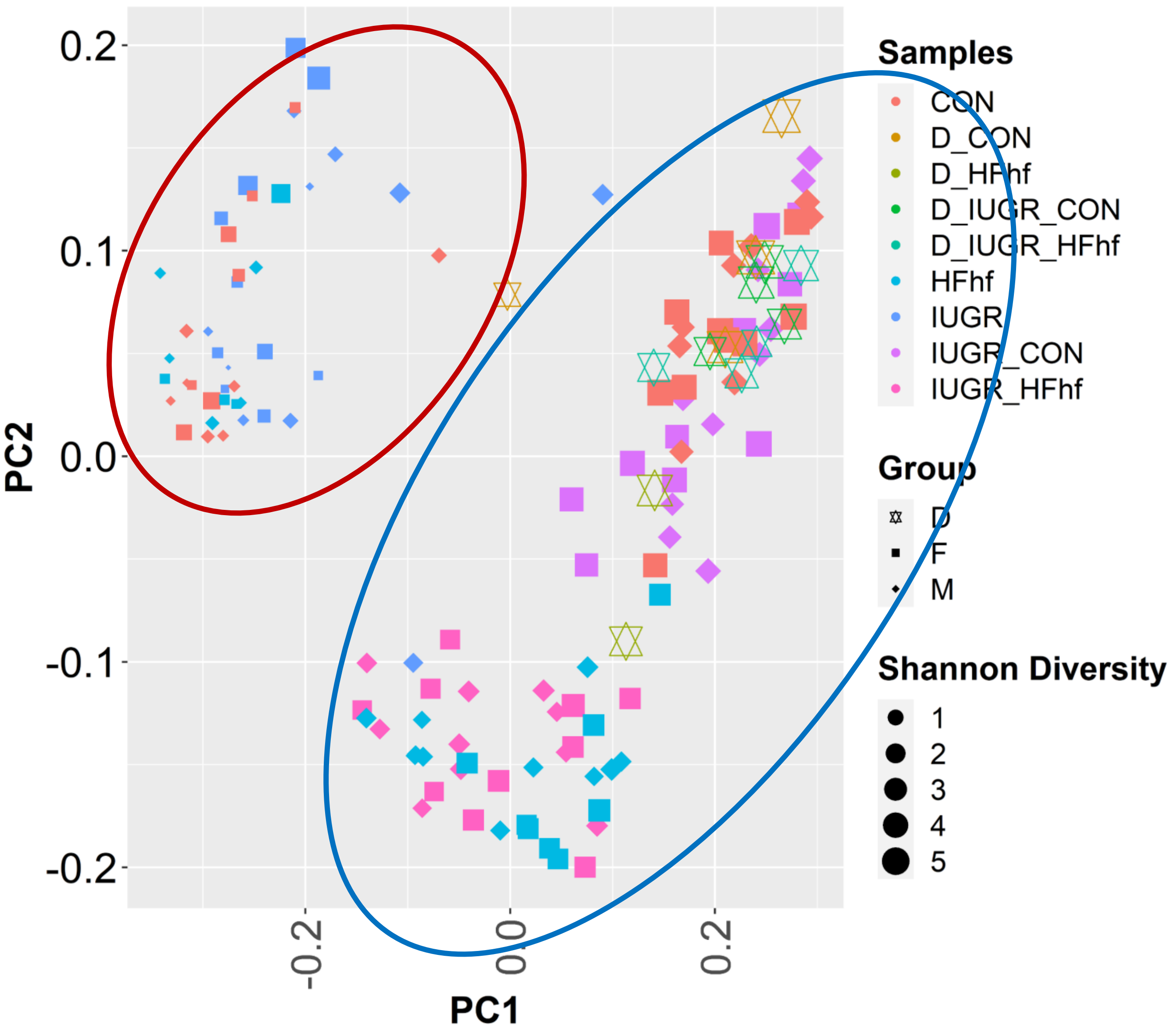

Beta diversity distribution along with Shannon diversity (alpha diversity) from PN2 and PN21 samples along with Dams . IUGR_CON is the same as IUGR-RC. D = dams, F = female, and M = male.

Supplementary Table 1

Group	Mothers	Pups	Litter size	SEM	Comparison	p-value
CON	4	47	11.8	0.48	CON vs IUGR	0.27
IUGR	8	103	12.9	0.4	CON vs HFhf	0.18
HFhf	4	41	10.3	0.63	IUGR vs HFhf	0.006

The total number of pups and the litter size (mean)±SEM at postnatal day 2 (PN2) arising from three experimental groups have been shown. The p values signify ANOVA followed by the Tukey test demonstrating the shown inter-group differences.

Supplementary Table 2

Post Natal (2Day) Differentially abundant phylum Rat Microbiome from gut/stool

Table with logFC for differentially abundant phyla

taxon_id	Intercept	HFhf	IUGR	Sex
Phylum:Firmicutes	4.200976	0	0	0
Phylum:Bacteroidetes	0	0	1.856192	0
Phylum:Proteobacteria	7.285953	0	0	0
Phylum:Fusobacteria	-1.70505	-0.41599	0	0
Phylum:Actinobacteria	2.747441	0	0	0
Phylum:Verrucomicrobia	0	0	1.969834	0

Table with Significance for differentially abundant phyla TRUE= significant FALSE = not-significant

Taxon_id	Intercept	HFhf	IUGR	Sex
Phylum:Firmicutes	TRUE	FALSE	FALSE	FALSE
Phylum:Bacteroidetes	FALSE	FALSE	TRUE	FALSE
Phylum:Proteobacteria	TRUE	FALSE	FALSE	FALSE
Phylum:Fusobacteria	TRUE	TRUE	FALSE	FALSE
Phylum:Actinobacteria	TRUE	FALSE	FALSE	FALSE
Phylum:Verrucomicrobia	FALSE	FALSE	TRUE	FALSE

Supplementary Table 3

Post Natal (21Day) Differentially abundant phylum Rat Microbiome from gut/stool

Table with logFC for differentially abundant phyla

taxon	(Intercept)	HFhf	IUGR_CON	IUGR_HFhf	Sex	Collection
**Phylum:Firmicutes**	3.581971931	-6.21E-05	0.04849005	0.016647661	0.056026	0.050350914
**Phylum:Bacteroidetes**	3.979572552	1.380783777	-0.219990483	0.669823626	-0.14778	-0.023237845
**Phylum:Cyanobacteria**	-2.700423613	-1.24251563	-0.705225624	-1.931157061	-0.15255	0.044469956
**Phylum:Tenericutes**	-2.650540091	-1.36943621	0.715576625	-1.106487264	0.52302	-0.321267602
**Phylum:Proteobacteria**	1.268688981	1.576624452	-0.020074387	1.311650042	0.296682	0.193720572
**Phylum:Deferribacteres**	-2.068136066	-2.4590527	-2.689409252	-3.003559785	0.531157	0.240992896
**Phylum:Actinobacteria**	-4.801385299	0.809693492	0.080150308	2.494134893	0.090873	-0.326646089
**Phylum:Verrucomicrobia**	2.547223186	1.059006999	0.629372524	2.05114295	-0.31527	-0.019742174

Table with Significance for differentially abundant phyla TRUE= significant FALSE = not-significant

taxon	(Intercept)	HFhf	IUGR_CON	IUGR_HFhf	Sex	Collection
**Phylum:Firmicutes**	TRUE	FALSE	FALSE	FALSE	FALSE	FALSE
**Phylum:Bacteroidetes**	TRUE	TRUE	FALSE	FALSE	FALSE	FALSE
**Phylum:Cyanobacteria**	TRUE	TRUE	FALSE	TRUE	FALSE	FALSE
**Phylum:Tenericutes**	TRUE	TRUE	FALSE	FALSE	FALSE	FALSE
**Phylum:Proteobacteria**	TRUE	TRUE	FALSE	TRUE	FALSE	FALSE
**Phylum:Deferribacteres**	TRUE	TRUE	TRUE	TRUE	FALSE	FALSE
**Phylum:Actinobacteria**	TRUE	TRUE	FALSE	TRUE	FALSE	FALSE
**Phylum:Verrucomicrobia**	TRUE	TRUE	FALSE	TRUE	FALSE	FALSE

No significant difference in phylum abundance based on sex or collection time

Supplementary Table 4

Adult (180 D) Differentially abundant phylum Rat Microbiome from gut/stool

Table with logFC for differentially abundant phyla

	Intercept	HFhf	IUGR_RC	IUGR_HF hf	Sex	Collection
**Phylum:Firmicutes**	7.902219	0.958868	0.960852	1.031536	-0.00657	-0.054506849
**Uncharectarized**	-1.53949	-0.61434	-0.08818	-0.66004	0.384251	0.17274403
**Phylum:Actinobacteria**	2.905742	-0.16139	0.349445	-0.01525	0.235036	0.112005905
**Phylum:Tenericutes**	3.71767	-4.94262	-0.09211	-3.04067	-0.16827	0.103450622
**Phylum:Proteobacteria**	2.791572	2.476539	-0.61468	2.09338	0.40849	-0.229234652
**Phylum:Deferribacteres**	0.000568	0.319229	-1.14113	0.056668	0.633027	0.120322729
**Phylum:Lentisphaerae**	-1.91064	0.053391	0.370421	0.065877	0.010824	-0.018280297
**Phylum:Cyanobacteria**	-0.9956	-0.5032	-0.43548	0.389315	-0.15952	-0.156663948
**Phylum:Verrucomicrobia**	4.083515	1.713566	-0.06416	2.050682	-0.33183	0.199423899
**Phylum:TM7**	-2.08105	0.458498	0.459738	0.854883	-0.07626	-0.003484103
**Phylum:Bacteroidetes**	6.829192	0.099684	-2.29599	0.058901	0.162351	0.064376957

Table with Significance for differentially abundant phyla
TRUE= significant FALSE = not-significant

	Intercept	HFhf	IUGR_RC	IUGR_HF hf	Sex	Collection
**Phylum:Firmicutes**	TRUE	TRUE	TRUE	TRUE	FALSE	FALSE
**Uncharectarized**	TRUE	TRUE	FALSE	TRUE	FALSE	FALSE
**Phylum:Actinobacteria**	TRUE	FALSE	FALSE	FALSE	FALSE	FALSE
**Phylum:Tenericutes**	TRUE	TRUE	FALSE	TRUE	FALSE	FALSE
**Phylum:Proteobacteria**	TRUE	TRUE	FALSE	TRUE	FALSE	FALSE
**Phylum:Deferribacteres**	FALSE	FALSE	FALSE	FALSE	FALSE	FALSE
**Phylum:Lentisphaerae**	TRUE	TRUE	TRUE	TRUE	FALSE	FALSE
**Phylum:Cyanobacteria**	TRUE	TRUE	TRUE	FALSE	FALSE	FALSE
**Phylum:Verrucomicrobia**	TRUE	TRUE	FALSE	TRUE	FALSE	FALSE
**Phylum:TM7**	TRUE	TRUE	TRUE	FALSE	FALSE	FALSE
**Phylum:Bacteroidetes**	TRUE	FALSE	TRUE	FALSE	FALSE	FALSE

No significant difference in phylum abundance based on sex or collection time
